# Hacia una justicia social y alimentaria de personas jornaleras agrícolas con migración interna en México

**DOI:** 10.1590/0102-311XES054424

**Published:** 2025-05-26

**Authors:** Ariadna Guadalupe Villalobos-Pérez, Maria de Lourdes Flores López, Florence L. Théodore, María Ángeles Villanueva-Borbolla, Kim Sánchez Saldaña

**Affiliations:** 1 Instituto Nacional de Salud Pública, Cuernavaca, México.; 2 Consejo Nacional de Humanidades, Ciencia y Tecnologías, Cuidad de México, México.; 3 Centro de Investigación y Asistencia en Tecnología y Diseño del Estado de Jalisco, Guadalaraja, México; 4 Universidad Autónoma del Estado de Morelos, Cuernavaca, México.

**Keywords:** Trabajadores Rurales, Derecho Humano a una Alimentación Adecuada, Política Nutricional, Migración Interna, Justicia Social, Rural Workers, Human Right to Adequate Food, Nutritional Policy, Internal Migration, Social Justice, Trabalhadores Rurais, Direito Humano à Alimentação Adequada, Política Nutricional, Migração Interna, Justiça Social

## Abstract

Los objetivos fueron caracterizar el ambiente alimentario de las personas jornaleras agrícolas con migración interna en México y construir escenarios en términos prospectivos para formular alternativas que orienten las políticas agroalimentarias desde un enfoque en justicia social. Se emplearon métodos cualitativos, incluidas entrevistas semi-estructuradas a mujeres jornaleras y especialistas en el tema, observación en campos de cultivo de chile en Guanajuato, captura de imágenes, así como talleres de prospectiva alimentaria. Los datos fueron analizados mediante análisis de contenido. Las personas jornaleras agrícolas con migración interna enfrentan desventajas sistemáticas en toda la ruta migratoria, inscripto en un contexto de alta vulnerabilidad y discriminación. Viven en situación de precariedad, tanto en el trabajo como en el acceso a alimentos adecuados, inmersos en un ambiente alimentario malsano, rodeado de desiertos y pantanos alimentarios. Un escenario plausible donde puedan migrar bajo condiciones justas y con garantía de sus derechos laborales y alimentarios. Las personas jornaleras agrícolas con migración interna no tienen cubiertas las seis dimensiones de justicia social para alcanzar el bienestar, reflejado en un ambiente alimentario que promueve injusticias, desigualdad y vulnerabilidad alimentaria. Se refuerza la necesidad urgente de políticas públicas orientadas a mejorar las condiciones laborales, de vida y de alimentación en áreas rurales a través del impulso de las economías locales, y buscar que la migración sea una opción y no una obligación. Implementar estas políticas no solo mejoraría su vida, sino que también promovería un modelo alimentario más justo para el campo mexicano, desde la soberanía alimentaria.

## Abstract (en)

This study aimed to characterize the food environment of internal agricultural migrant workers in Mexico and to build scenarios in prospective terms to formulate alternatives that guide food and agriculture policies based on a social justice approach. Qualitative methods were used, including semi-structured interviews with women who are laborers and specialists in the subject, observation in chili cultivation fields in Guanajuato, image capture, as well as food foresight workshops. The data were analyzed via content analysis. Internal agricultural migrant workers face systematic disadvantages throughout migration, inscribed in a context of high vulnerability and discrimination. They live in a precarious situation, both in terms of work and access to adequate food, immersed in an unhealthy food environment, surrounded by deserts and food swamps. A plausible scenario in which they can migrate under fair conditions and with guarantees of their labor and food rights. Internal agricultural migrant workers’ six dimensions of social justice to achieve well-being are not covered, reflected in a food environment that promotes injustices, inequality, and food vulnerability. The acute need for public policies aimed at improving working, living, and food conditions in rural areas by promoting local economies, and seeking to make migration an option and not an obligation, is reinforced. Implementing such policies would not only improve their lives but also promote a fairer food model for the Mexican countryside, based on food sovereignty.

## Resumo

Os objetivos deste estudo foram identificar o ambiente alimentar de trabalhadoras agrícolas diaristas em migração interna no México e construir cenários prospectivos para formular alternativas que orientem as políticas agroalimentares a partir da abordagem da justiça social. Foram utilizados métodos qualitativos, incluindo entrevistas semiestruturadas com trabalhadoras diaristas e especialistas no assunto, observação em cultivos de pimenta em Guanajuato, captura de imagens, além de oficinas de prospecção alimentar. Os dados foram analisados por meio de uma análise de conteúdo. As trabalhadoras agrícolas diaristas em migração interna enfrentam desvantagens sistemáticas ao longo da rota de migração em um contexto de alta vulnerabilidade e discriminação. Vivem em situação precária, tanto em relação ao trabalho quanto no acesso à alimentação adequada, imersas em um ambiente alimentar insalubre e cercado por desertos e pântanos alimentares. Um cenário viável onde possam migrar em condições justas e com seus direitos trabalhistas e alimentares garantidos. As trabalhadoras agrícolas diaristas em migração interna não têm atendidas as seis dimensões da justiça social para alcançar o bem-estar, refletindo em um ambiente alimentar que promove injustiças, desigualdades e vulnerabilidade alimentar. Reforça-se a necessidade urgente de políticas públicas que visem à melhoria das condições de trabalho, de vida e de alimentação nas zonas rurais por meio da promoção das economias locais, que tornam a migração uma opção e não uma obrigação. A implementação dessas políticas não só melhoraria suas vidas, mas também promoveria um modelo alimentar mais justo para a zona rural do México, com base na soberania alimentar.

## Introducción

Se estima que en el mundo el número de personas trabajadoras agrícolas constituyen aproximadamente una tercera parte de la fuerza de trabajo, concentradas en países de medianos y bajos ingresos ^1^. Experimentan condiciones análogas a la esclavitud debido a que se integran a un modelo alimentario basado en el agronegocio, descrito por Otero ^2^ como “régimen alimentario neoliberal”. Se caracteriza por prescindir del bienestar del trabajador, la liberación comercial y la neoregulación agrícola con leyes a favor de agroempresas multinacionales. Este modelo ha propiciado desigualdades económicas y sociales, comercialización masiva de comestibles ultraprocesados, hambre, devastación ambiental, uso de transgénicos, agrotóxicos, pérdida de cultura y del sustento rural ^2^ ^,^ ^3^.

En México, a partir de la instauración de este régimen la autosuficiencia alimentaria del país empezó a declinar, agudizándose con la firma del Tratado de Libre Comercio con Estados Unidos y Canadá (1994-2008) ^4^, lo que afectó profundamente a los productores rurales que se vieron en desigualdad de condiciones para continuar con la producción agrícola, a la vez que las agroempresas multinacionales empezaron a controlar el mercado nacional al amparo del Estado. Esto trajo consigo el despojo de tierras y el desplazamiento de campesinos a campos de explotación agroindustrial para emplearse como jornaleros agrícolas temporales, quienes migran de sus lugares de origen (Sureste de México en zonas con altos índices de pobreza y con mayor proporción de población indígena) hacia zonas de cultivo intensivo de exportación (Centro y Norte), donde son sometidos a condiciones de explotación y precariedad laboral ^5^ ^,^ ^6^ ^,^ ^7^.

En 2022, la población jornalera agrícola a nivel nacional era de 2,3 millones de las cuales el 60,5% se encontraba en situación de pobreza ^8^. A pesar de que desempeñan un papel fundamental en la producción de alimentos frescos, a nivel local el 80% presentaba carencia por acceso a la alimentación ^8^. Guanajuato es el séptimo estado con mayor producción agrícola, con una superficie sembrada de 678,37 hectáreas ^9^, por lo que se vuelve un estado destino para población jornalera que migra desde el sur y a su vez alberga población jornalera local.

La vulnerabilidad alimentaria se expresa en la disminución de prácticas de autoconsumo y un aumento en la adquisición de comestibles ultraprocesados ^10^. Situación que está determinada por el ambiente alimentario y que aumenta la doble carga de la mala nutrición, inseguridad alimentaria, enfermedades infecciosas recurrentes y enfermedades crónicas no transmisibles (ECNT) ^11^ ^,^ ^12^ ^,^ ^13^. 

El ambiente alimentario es crucial, abarca las dimensiones socioculturales, políticas y económicas que configuran la interacción de las personas con el sistema alimentario y permite entender cómo el entorno moldea sus elecciones alimentarias ^14^. La baja accesibilidad y disponibilidad de alimentos en zonas de trabajo hace que la población jornalera enfrente desafíos en materia de alimentación, como la vulneración constante de su derecho humano a la alimentación, el cual se ejerce cuando toda persona tiene acceso físico y económico, en todo momento, a una alimentación adecuada en calidad y cantidad y a los medios para obtenerla ^15^. 

Las estrategias pasadas, destinadas a mejorar sus condiciones de vida, han sido asistencialistas y desarticuladas entre las diferentes dependencias gubernamentales, y superficiales al no abordar las causas estructurales para mitigar los efectos de las políticas neoliberales ^16^. Además, su atención ha desaparecido de la agenda pública, con la eliminación en 2018 del Programa de Apoyo a Trabajadores Agrícolas (PAJA), que si bien no resolvía la problemática en su totalidad, les representaba una ayuda importante ^6^. 

Ante el ausente papel del Estado, la academia y las organizaciones de la sociedad civil han apelado al derecho a un trabajo digno, señalando las violaciones sobre los derechos humanos que enfrentan. Resulta relevante abogar por el derecho humano a la alimentación con vistas a la mejora de su bienestar, mismo que está reconocido en el artículo 4º de la Constitución Política de los Estados Unidos Mexicanos.

Frente a esta realidad, se planteó como objetivo caracterizar el ambiente alimentario de las personas jornaleras agrícolas con migración interna en México, junto con sus condiciones de trabajo para construir escenarios en términos prospectivos con el propósito de formular alternativas que orienten las políticas agroalimentarias en México, desde un enfoque en justicia social.

## Material y métodos 

### Marco conceptual

A partir de un diagnóstico, abordamos las problemáticas que enfrentan las personas jornaleras agrícolas con migración interna en México, así como la caracterización de su ambiente alimentario. Analizamos las desventajas sistemáticas que obstaculizan el logro del bienestar social y la plena realización del derecho humano a la alimentación. 

De manera sucinta, el concepto de ambiente alimentario establece que las elecciones de estilo de vida están moldeadas por una compleja interacción de factores altamente interrelacionados. Cerda Rioseco et al. ^17^ abordan el ambiente alimentario desde una perspectiva socioecológica, considerando cinco dimensiones interrelacionadas: (1) doméstico, espacio primario de socialización (viviendas); (2) institucional, lugar donde se venden o proporcionan alimentos en el espacio de trabajo (campos); (3) vía pública, aborda la comercialización de alimentos en espacios públicos (calle); (4) restauración, espacios de consumo de alimentos fuera del hogar (fondas o restaurantes); y (5) abastecimiento, espacios donde se adquieren alimentos (mercados, tienda de abarrotes o supermercados) y condiciona su disponibilidad y accesibilidad. 

Se utilizó la prospectiva ^18^ como propuesta de cambio, mediante la construcción de escenarios futuros con la participación de actores clave. Lo que permitió proponer y planificar acciones conjuntas desde diversos ámbitos a corto, mediano y largo plazo, con potencial para impulsar la generación de soluciones para el alcance del bienestar social y el cumplimiento del derecho humano a la alimentación. 

### Diseño del estudio

La población de estudio estuvo compuesta por personas jornaleras agrícolas en contexto de migración temporal en Guanajuato y pertenecientes a una cultura indígena. A partir de metodología cualitativa, el trabajo de campo se realizó durante la temporada de cosecha agrícola de chile jalapeño y serrano en campos agrícolas y viviendas temporales localizadas en la periferia de las localidades de León, Silao, Romita, San Francisco del Rincón, Manuel Doblado y Purísima del Rincón (abril-mayo 2023). La incorporación de participantes se realizó a través de muestreo intencional y bola de nieve en coordinación con una organización de la sociedad civil que brinda acompañamiento a persona jornalera agrícola con migración interna en el estado. 

El estudio comprendió dos componentes: (1) un diagnóstico con la caracterización de las personas jornaleras agrícolas con migración interna y ambiente alimentario, y (2) la aplicación de prospectiva con actores clave. Se utilizaron los siguientes instrumentos: 

(a) Observación no participante y registro fotográfico: se realizaron visitas a diez campos de cultivo en seis municipios, tres viviendas, dos tiendas de comida y observación de puntos de venta;

(b) Entrevistas semiestructuradas: se realizaron ocho entrevistas a actores clave (2 mujeres jornaleras, 2 activistas, 2 investigadoras y 2 legisladores) sobre las problemáticas que enfrentan las personas jornaleras agrícolas con migración interna, su ambiente alimentario y aspectos prospectivos. Las entrevistas fueron grabadas con previo consentimiento y se mantuvo la confidencialidad de los informantes. 

(c) Escenarios y prospectiva: con el propósito de construir posibilidades sobre el futuro alimentario para las personas jornaleras agrícolas con migración interna, se llevó a cabo un taller presencial con 28 personas que acompañan a la población en los campos de Guanajuato desde hace más de 10 años. Dentro de los participantes había nutriólogas, pedagogas, defensores de derechos humanos, abogados, comunicólogas, entre otros. El taller exploró el contexto actual y la generación de escenarios ideales y plausibles. Los participantes propusieron acciones de cambio y políticas impulsoras para lograr los escenarios planteados, situando actores, acciones, alianzas, recursos y temporalidad. 

Adicionalmente, con el propósito de comprender la percepción de la niñez jornalera, se llevaron a cabo dos actividades lúdicas relacionadas al consumo y preferencias alimentarias con niños y niñas jornaleros de 6 a 12 años dentro de los campos como parte de las actividades de la organización de la sociedad civil. 

Se realizó una transcripción detallada de las entrevistas y a partir de la lectura repetida de los datos empíricos, se codificó y categorizó la información en el programa Microsoft Excel (https://products.office.com/). En las actividades de prospectiva, se realizó una relatoría, la transcripción de los audios y la sistematización del material elaborado. En ambos instrumentos se analizaron las narrativas discursivas de los participantes, considerando los ejes transversales de la investigación mediante análisis de contenido.

Para profundizar en la accesibilidad física a los alimentos se aplicó la metodología de Reyes-Puente et al. ^14^. Se elaboró una representación gráfica de la distancia (km) del centro de cada campo hacia la tienda de abarrotes más cercana con el software de Sistemas de Información Geográfica Quantum (QGIS; https://qgis.org/en/site/) y el Directorio Estadístico Nacional de Unidades Económicas (DENUE) ^19^. 

Finalmente, se llevó a cabo la triangulación de toda la información recabada, lo que permitió la construcción de cuatro escenarios (catastrófico, posible, ideal y plausible) basados en la perspectiva de los informantes clave. Estos escenarios se utilizaron como base para la formulación de recomendaciones finales. El estudio fue aprobado por el Comité de Ética del Instituto Nacional de Salud Pública (Proyecto CI1314, folio identificador G88) y se obtuvieron todos los consentimientos informados.

## Resultados

### Diagnóstico: caracterización de las personas jornalera

Las personas jornaleras agrícolas con migración interna trabajan en campos de cultivo de medianos y grandes productores agroindustriales de Guanajuato. Durante las entrevistas, los participantes señalaron desconocer las empresas a las que destina el chile. Sin embargo, se conoce que, los primeros cortes de chile son trasladados al norte del país, por lo que pueden ser de exportación, mientras que los últimos cortes se distribuyen localmente. Se observó una reserva significativa respecto al nombre de las empresas, lo cual limita el acceso a información sobre estas corporaciones agroindustriales.

Los migrantes pertenecen al pueblo indígena Ñuu Savi (Mixtecos), siendo principalmente originarios de Guerrero (Región de la Montaña) y una pequeña proporción de Oaxaca (Mixteca Oaxaqueña). En menor medida hay familias jornaleras del pueblo Me’phaa (tlapanecos) y Náhuatl. Mantienen su vestimenta, tradiciones e idioma, en particular algunas mujeres son monolingües o hablan poco castellano. La mayoría hace migración interna dentro del territorio mexicano. No obstante, algunos integrantes (principalmente hombres jóvenes) migran a Estados Unidos en búsqueda de mejores condiciones de vida.

La contratación por parte del empresario agrícola se hace de manera informal mediante un intermediario llamado “caporal” (hombre), quien es el responsable de formar cuadrillas mediante el reclutamiento de personas jornaleras agrícolas con migración interna. Su papel es crucial para la comunicación con el empleador, la supervisión y distribución del trabajo, y fungir como intermediario en el pago. El número de cuadrillas contratadas varía de acuerdo con el empleador y la extensión de cultivo. La mayoría realiza una migración familiar, ya sea con familia nuclear u otras configuraciones familiares donde predominan lazos consanguíneos, llevando consigo a sus hijos e hijas, quienes se insertan en el trabajo jornalero desde edades tempranas (8-10 años) para incrementar el ingreso económico. 

Después de la temporada de pizca en Guanajuato (abril-junio), los ciclos laborales no son los mismos para todos, algunos siguen la migración a Jalisco, Colima, San Luis Potosí y Sinaloa, donde continúan trabajando entre cuatro y seis meses dependiendo la oferta laboral, para finalmente regresar a sus comunidades. Sin embargo, algunas familias ya no regresan y se han asentado en esos estados.

A lo largo de la ruta migratoria y en los lugares de destino se encuentran con diversas problemáticas (Cuadro 1), empezando en sus lugares de origen, donde migran de manera forzada dada las condiciones de pobreza y violencia estructural. En el traslado viajan en condiciones de hacinamiento y en los lugares destino, dependiendo del empleador y ciudad, estas pueden exacerbarse enfrentando continuas violaciones a sus derechos: 

**Cuadro 1:** Problemáticas en la ruta migratoria de la población jornalera agrícola con migración interna en México.

Problemáticas por la actual situación de la ruta migratoria	Lugares de origen: - Desplazamiento interno forzado por: violencia, megaproyectos, llegada del crimen organizado, falta de oportunidades laborales, carencias sociales, despojo de tierras y comunidades olvidadas. Traslados: - Camionetas descuidadas y sobresaturadas, largas horas de hacinamiento, inseguridad, accidentes viales, extorsión constante por parte de autoridades viales. Lugares de destino: - Falta de condiciones laborales dignas: jornadas de explotación (12-16 horas de trabajo) bajo condiciones climáticas severas, trabajo a destajo (arpillas por día), incertidumbre laboral, sin días de descanso, bajos salarios (MXN 18-MXN 25 por arpilla), sin contrato laboral, sin seguridad social, campos sin baños y agua. - Falta de condiciones de vivienda digna: no cuentan con servicios básicos, viven en hacinamiento y marginación, falta de acceso a agua potable y corriente, sin protección ante el clima, no hay albergues. - Enfermedades: de tipo infecciosas y crónico degenerativas, accidentes laborales, intoxicación por agroquímicos y muerte infantil. - Discriminación y pleitos con las comunidades de destino. - Falta de espacios para la niñez: esperan a la orilla del surco en camionetas sin supervisión y condiciones precarias. - Niñez trabajadora o cuidadora: son responsables de sus hermanos menores (niñas) o se unen al trabajo familiar a temprana edad.
Problemáticas estructurales	- Difícil acceso a servicios de salud y seguridad social, los cuales criminalizan y discriminan. - Sin acceso a una educación continua e intercultural. - Falta de traductores, mujeres monolingües. - Inseguridad alimentaria y doble carga de la mala nutrición. - Violencia sistémica, estructural y de género. - Pobreza. - Discriminación. - Invisibilización por parte de las autoridades y de la sociedad.
Testimonios	“*A veces los domingos nos dan un día, ya sí empieza duro el trabajo todos los días, no tenemos horario de entrar y salir porque pues según las arpillas que nos den, y a veces andamos ya casi terminado cuando está casi oscureciendo y pues le tenemos que seguir*” (jornalera A, Guerrero). “*La lleve a ella* [su mamá] *al doctor y me regañaron mucho que porque no la lleve, y le dije: es que no tengo como llevarla*” (jornalera E, Guerrero). “*Las comunidades cercanas son discriminatorios con ellos, ni siquiera les rentan los espacios, solo se las rentan en obra negra y cuando la arreglan con el dinero de los jornaleros al año quentra ya no se las quieren rentar*” (miembro de organización de la sociedad civil, Guanajuato). “*Hay una deuda histórica todavía muy grande con ellos, y te puedo decir: hoy están trabajando en condiciones de esclavitud sin ningún derecho mínimo social*” (legisladora, Tlaxcala).

Fuente: elaboración propia.

“*No hay acceso a la educación, salud, alimentación y esta periferia hace que el mismo recorrido y condiciones que tienen para moverse, cocinar, sea más difícil de acceder… pareciera que son como eslabones que van dificultando el acceso a derechos y que incluso muchas vece no les interesa las vidas de las personas jornaleras, y les dejan morir*” (mujer E, miembro de organización de la sociedad civil, Guanajuato).

### Caracterización del ambiente alimentario

El ambiente alimentario varía de acuerdo con el estado de la ruta migratoria, el tipo de empleador y el lugar en el que se establezcan temporalmente. Un señalamiento que se repitió en entrevistas con mujeres jornaleras fue que hay un cambio en el consumo de alimentos que está mediado por las condiciones en los campos, la disponibilidad, la accesibilidad física y económica, la aceptabilidad y el tiempo, tal expresado por una jornalera:

“*Allá en Sinaloa hay muchas cosas que comer, allá es puro mar, a veces así hay muchas cosas, hay muchas mojarras, hay mucho camarón está muy barato, cuando nosotros venimos aquí* [Guanajuato] *pues no hay pescado, ni camarones, no podemos conseguir, y pues lo que haiga comemos* (...) *El pollo que hay aquí casi no nos gusta porque son puros pollos pintados y allá* [Jalisco] *es como pollo de granja y de esos son los que nos gusta* (...) *Cuando andamos aquí pues ni tiempo nos da de ir a comprar y por eso a veces llegamos y no tenemos que preparar pa’ comer, y ya, aunque sea una salsita así hacemos*” (jornalera A, Guerrero).

Dentro de la dieta que llevan en sus comunidades, se destacan aspectos de soberanía alimentaria, como el uso del sistema milpa, la agricultura familiar, la crianza de aves de corral para autoconsumo y la relación con el campo. Sin embargo, el consumo de comestibles ultraprocesados y la inseguridad alimentaria es recurrente. Así lo expresó un niño: 

Niño: “*En Guerrero come hierba, hasta con tierra come cuando no hay comida*”

Facilitadora: “*¿Qué tienen allá?*”

Niño: “*Tierra para sembrar*”

Facilitadora: “*¿Qué siembran?*”

Niño: “...*elote, calabaza, chile, semilla de frijol, cebolla, tomate de cáscara, maíz morado*” (niño jornalero, Guerrero).

**Figura 1:**
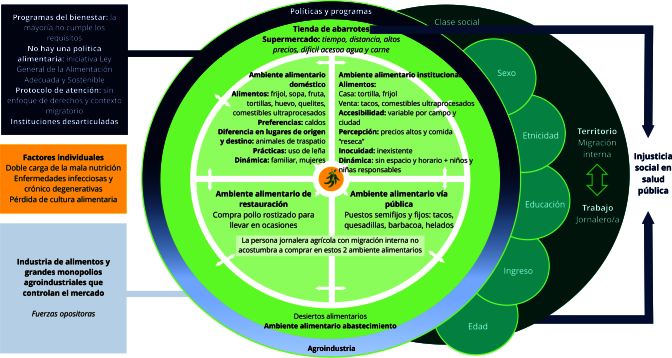
Modelo gráfico del ambiente alimentario de personas jornaleras agrícolas con migración interna.

En la Figura 1, se muestra el modelo de los cinco ambientes alimentarios interrelacionados de las personas jornaleras agrícolas con migración interna durante su estancia migratoria en Guanajuato, así como los componentes estructurales y determinantes sociales que los condicionan. Destacan el territorio y el trabajo, debido a la naturaleza de su ocupación. También se ilustran las políticas y programas dirigidos a las personas jornaleras agrícolas con migración interna con su contraparte, la agroindustria. 

Fuente: elaboración propia, adaptado del modelo sobre ambientes alimentarios de chile (Cerda Rioseco et al. ^17^).

#### Ambiente alimentario doméstico

Generalmente, la vivienda no es proporcionada por el empleador, y deben rentar viviendas temporales caracterizadas por ser casas en obra negra o bodegas donde viven varias familias en hacinamiento separadas por lonas o cobijas. Están ubicadas dentro de localidades cercanas a los campos y no cuentan con servicios básicos. Las mujeres son encargadas de cocinar para la familia en estufas improvisadas de leña y el comal es un utensilio imprescindible para hacer tortillas. Varias familias tienen animales de traspatio como gallinas como una práctica regular. La alimentación consiste en una combinación limitada de una dieta rural basada en el maíz, frijol y hierbas silvestres que recolectan de los campos, mezclada con altas cantidades de comestibles ultraprocesados que tienen disponibles en tiendas cercanas, tal no narran las mujeres:

“*Hacemos diario tortilla con la leña y la comida nomás así de huevo o cositas así rápido lo hago en la estufa, pero, ya cuando hago frijol o cosas que duran más ya lo hago en la leña*” (jornalera A, Guerrero).

“*Antes de ir a trabajar comemos, llegamos en la tarde y comemos sopa instantánea, a veces salsa, a veces hacemos arroz* (...) *nosotros tomamos coca* (...) *Cuando no me gusta el pollo ni nada, yo voy* [a] *cortar hierba mora o quelites*” (jornalera E, Guerrero). 

#### Ambiente alimentario institucional

**Figura 2:**
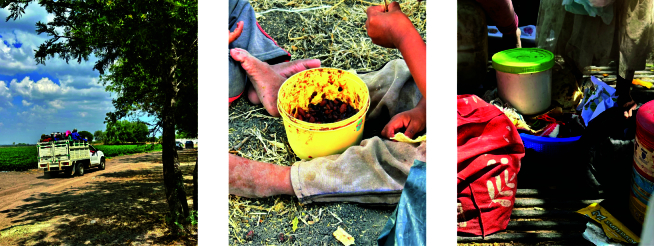
Alimentos consumidos por personas jornaleras agrícolas dentro del ambiente alimentario institucional. Guanajuato, México.

Dentro de los campos agrícolas no hay espacios dignos para comer, horarios de comida o comedores, ni acceso a agua potable. En la Figura 2 se observan fotografías referentes a este ambiente alimentario, donde los alimentos se consumen dentro del surco, bajo el rayo del sol o en las camionetas. Se identificaron dos escenarios: (1) la única fuente de alimentación proviene de lo llevado por las familias desde sus hogares, la cual se reduce al consumo de tortillas, frijol, pasta, chile y comestibles ultraprocesados; (2) cuando hay venta de alimentos dentro del campo (tacos de carne asada o dorados, frituras, refresco y helados), sin embargo, la venta no sucede en todos los campos. La percepción es que la comida que se vende es desabrida y tiene un alto costo, sin embargo, es su única opción:

Fuente: fotografías propias tomadas en campos de cultivo en Guanajuato, México.

“*Pues traen la comida así resequita... yo por mi pienso pues está bien* [que vendan]*, porque si nosotros no traemos comida una vez, pues ya de aquí compramos. Y yo digo pues donde no entran vendedores pues está difícil pa’ nosotros porque pues no podemos comprar*” (jornalera A, Guerrero). 

“[La comida] *está cara porque el otro año venimos y costaba a $15 un taco, pero ahora está como a $17*” (jornalera B, Guerrero).

**Figura 3:**
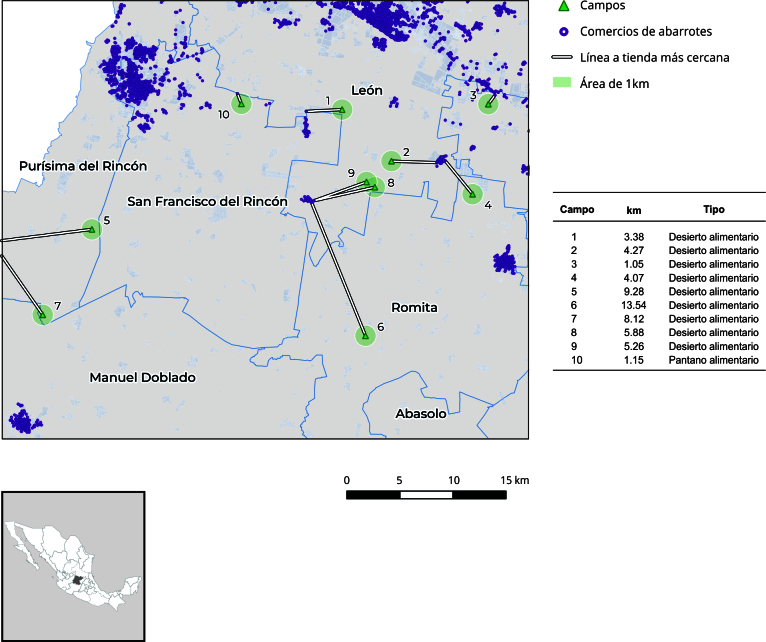
Mapeo del ambiente alimentario institucional de personas jornaleras agrícolas en campos de cultivo de chile. Guanajuato, México.

En la Figura 3, se observa la distancia desde los campos a la tienda de abarrotes más cercana, lo que evidencia la falta de acceso a alimentos dentro del trabajo. Se encontraron 8 desiertos alimentarios y 2 pantanos alimentarios en los campos visitados, que representan una opción de compra. 

Fuente: elaboración propia a partir del recorrido de los autores y datos del Instituto Nacional de Estadística y Geografía ^19^.

#### Ambiente alimentario de restauración y vía pública

A pesar de que en los caminos y localidades aledañas a los campos y viviendas hay establecimientos de venta de comida, la población no asiste a este tipo de establecimientos por falta de tiempo y dinero. Únicamente se mencionó la compra de pollo asado, ocasionalmente, los fines de semana.

#### Ambiente alimentario de abastecimiento

El principal lugar de abasto son las tiendas de abarrotes (1 a 3km de las viviendas), sin embargo, sus precios son considerados altos para las personas jornaleras agrícolas con migración interna. Algunas familias en los pocos días libres se organizan para ir a localidades cercanas como Romita (15 a 25km de distancia) donde hay supermercados/mercados con precios más accesibles. No obstante, la jornada laboral y la falta de días de descanso lo imposibilita. Consideran que lo más difícil de conseguir a un buen precio es el agua y el maíz, indispensables para su alimentación:

“*Aquí* [Guanajuato]*, lo que nosotros batallamos más y sufrimos más es del agua, porque aquí está muy caro. Y luego nosotros a veces nos acostumbramos a tomar agua del que saca así los pozos, pero nos pega diarrea con esa agua… Pasamos a las tiendas* [a comprar]*, pero están muy caras las cosas. Solo la MASECA* [harina de maíz nixtamalizado] *está a $250 un bulto y en otros lados ahora que venimos de Jalisco está a $200*” (Jornalera A, Guerrero).

### Escenarios y prospectiva

A partir de la voz de los participantes en este estudio, se construyeron cuatro escenarios descritos en el Cuadro 2. 

**Cuadro 2:** Escenarios planteados para las personas jornaleras agrícolas con migración interna en México.

ESCENARIOS	NOMBRE DE LOS ESCENARIOS	PROSPECTIVA : ¿CÓMO? POLÍTICAS Y ACCIONES IMPULSORAS	PROSPECTIVA: ¿QUIÉN?	PLAZO
Catastrófico (actual)	Las personas jornaleras agrícolas com migración interna son migrantes dentro de territorio mexicano, viven y trabajan bajo condiciones de precariedad laboral, injusticia alimentaria, y dentro de un sistema alimentario agroindustrial insostenible			
Probable	Nuevas generaciones de jornaleros y jornaleras siguen viviendo bajo el mismo sistema, aumenta la doble carga de mala nutrición y enfermedades			
Deseable (ideal)	Las personas jornaleras agrícolas com migración interna son campesinos, viven en sus comunidades de origen con una vida digna y soberanía alimentaria	- Garantizarles tierra y mercado	Garantiza el Estado y garantiza la sociedad	Largo plazo
		- Cambio en el modelo agrícola		
		- Fortalecimiento de sistemas de circuitos locales de producción		
		- Consumidores conscientes		
Plausible	Las personas jornaleras agrícolas com migración interna tienen acceso a una alimentación adecuada y culturalmente aceptada en los campos donde trabajan	- Comedores móviles y accesibles dentro de los campos de trabajo que impulsen prácticas de reciprocidad, con alimentos suficientes de acuerdo con las necesidades nutricionales por grupo etario y culturalmente aceptables	Garantiza el empleador junto con el Estado + comunidad	Corto plazo
	Existe una política alimentaria que garantice el derecho humano a la alimentación a grupos prioritarios como la población jornalera	- Expedición del reglamento a la Ley General de Alimentación Adecuada y Sostenible con enfoque intercultural, de género y protección del interés superior de la niñez. Así como las consideraciones a grupos prioritarios.	Garantiza el Estado a través del poder ejecutivo (STPS, SS, SB, SADER, INEGI, CONAPO)	Corto plazo
		- Políticas que fomenten la autosuficiencia alimentaria, mediante el fortalecimiento del sistema agroproductivo de traspatio y cadenas cortas de comercialización		Mediano plazo
		- Diagnóstico y censo actualizado de la situación y necesidades específicas de la persona jornalera agrícola com migración interna		Mediano plazo
	Las personas jornaleras agrícolas com migración interna realizan migración interna, en condiciones dignas y con el cumplimiento de sus derechos laborales, de vivienda, educación, salud y alimentación	- Garantizar el cumplimiento de los derechos laborales de las personas jornaleras agrícolas com migración interna mediante regulación e inspección sin criminalización hacia las familias	Garantiza el empleador y supervisa STPS. SADER, DIF, STPS, SIPINNA	Largo plazo
		- Generación de programas sociales dirigidos específicamente a las personas jornaleras agrícolas com migración interna que consideren las condiciones de migración temporal, con enfoque intercultural, de género y de la protección del interés superior de la niñez trabajadora		Mediano plazo
	La producción agropecuaria se realiza en las comunidades rurales, movilizando la economía local y existen regulaciones para la agroindustria basada en el bien común	- La sociedad se involucra y es consciente con la producción de alimentos, por lo que procura consumir localmente	Sociedad	Mediano plazo
		- Fomentar estudios para identificar a los actores de la cadena de suministro a nivel local, estatal y nacional, libres de conflicto de interés. Así, como investigación en nuevos modelos de producción autosuficientes y sostenibles, libres de agroquímicos	Garantiza el Estado mediante la SECTEI	Largo plazo

CONAPO: Consejo Nacional de Población; DIF: Sistema Nacional para el Desarrollo Integral de la Familia; INEGI: Instituto Nacional de Estadística y Geografía; SADER: Secretaría de Agricultura y Desarrollo Rural; SB: Secretaría del Bienestar; SECTEI: Secretaría de Educación, Ciencia, Tecnología e Innovación; SIPINNA: Secretaría Ejecutiva del Sistema Nacional de Protección de Niñas, Niños y Adolescentes; SS: Secretaría de Salud; STPS: Secretaría de Trabajo y Previsión Social.

Fuente: elaboración propia.

El escenario catastrófico describe el contexto actual en el que viven las personas jornaleras agrícolas con migración interna. La precariedad laboral y el desinterés por parte de los empleadores, así como el nulo papel del Estado para garantizar condiciones de vida, son parte de este escenario:

“*Nos ha tocado ver que cuando están trabajando las familias, entran máquinas a fumigar* (...) *hemos hablado con los ingenieros, pero nos dicen que no les pasa nada*” (activista, Guanajuato).

Para el escenario deseable, la mayoría coincidió en que lo “ideal” sería que las personas jornaleras agrícolas con migración interna no tuvieran que migrar. Que se les garantizaran condiciones de vida digna en sus lugares de origen, empleos, apoyo y beneficios derivados del trabajo agrícola, educación de calidad, servicios de vivienda, así como que se retomara la soberanía alimentaria del país: 

“*¿Lo ideal es no migrar o libremente migrar?* (...) *Como decía Tlachinollan* [Migrar o morir]*, entonces si tuvieran una gama de posibilidades migrar sería una decisión... y muchas veces en esta población es por obligación*” (hombre V, miembro de organización de la sociedad civil, Guanajuato).

“*El ideal sería que no dependieran de los patrones para alimentarse, que el cultivo de las tierras sea destinado a su propia alimentación y va de la mano de la soberanía alimentaria, el dejar de ver a los alimentos como una mercancía y que sean la fuente de vida*” (mujer E, miembro de organización de la sociedad civil, Guanajuato).

Finalmente, para el escenario plausible, a través de la implementación de acciones a corto y mediano plazo, se consideró la migración interna en condiciones dignas, contratos laborales adecuados que garanticen salarios dignos, jornadas laborales conforme a la ley, horario para consumir alimentos y que la persona jornalera agrícola con migración interna sea sujeta de derechos contemplando sus necesidades. A un largo plazo, la reestructuración del sistema alimentario para asegurar condiciones de vida digna en sus hogares, en vista al logro del escenario ideal previamente descripto: 

“*Partiría primero de saber ¿qué les gusta?, ¿qué podrían probar? Y pensar en contar con este espacio que tenga algo sencillo, aislado de la zona donde están habituados, que fuera una infraestructura que contará con un centro de lavado, con mesas para que ellos puedan llegar, sentarse y probar los alimentos*” (investigadora, Morelos).

## Discusión

Los resultados del diagnóstico de la persona jornalera agrícola con migración interna, mediante la caracterización del contexto y ambiente alimentario, revelan una clara violación al derecho humano a la alimentación que impide el alcance del bienestar social. Como lo han documentado anteriormente Powers & Faden ^20^, la justicia social involucra dimensiones del bienestar que garantizan que toda persona disponga de una base material, cultural y política suficiente para el logro de una vida digna. Por ello analizamos las desventajas sistemáticas que enfrentan las personas jornaleras agrícolas con migración interna inscriptas en un contexto de alta vulnerabilidad y discriminación que empieza desde sus comunidades de origen, pero que se agudiza durante los traslados y en los Estados a donde migran, caracterizados por la precariedad laboral y un ambiente alimentario injusto. 

Partimos de las injusticias estructurales que viven día a día, tales como discriminación, pobreza y violencia (social y de género), que conllevan un daño a su integridad. Esta situación se relaciona con todas las dimensiones de la justicia social, donde se sustenta que las injusticias estructurales implican un perjuicio integral para la persona. 

Estas injusticias dan inicio desde sus lugares de origen donde experimentan condiciones que restringen la toma de decisiones y de su desarrollo, ya que son provenientes de los estados con mayor pobreza por ingreso en el país (Guerrero 66,4% y Oaxaca 61,7%) y con mayor proporción de población indígena ^21^. Las personas jornaleras agrícolas con migración interna, viven con una serie de carencias sociales que los obligan a migrar, como la falta de acceso a seguridad social (87,6%), a servicios de salud (67,7%), a servicios básicos de vivienda (54,1%), rezago educativo (52,5%), acceso a alimentos (30,6%), y carencia de vivienda digna (22,8%) ^8^.

Al llegar a los lugares de destino, son víctimas de rechazo por parte de los locales, lo que les imposibilita la generación de lazos. Adicionalmente, debido a la migración temporal y largas jornadas, el tiempo libre para crear tejidos sociales con personas fuera de lo familiar es limitado y, por lo tanto, impide el alcance de la dimensión de apego. Esta situación es similar a lo observado por Pérez-Soto et al. ^22^, donde la afiliación se da solo con los integrantes de su familia y con compañeros de trabajo, limitando la generación de redes de apoyo comunitarias. 

La exposición a riesgos laborales junto con el difícil acceso a servicios de salud y alimentación adecuada da como resultado un incremento de enfermedades, accidentes, intoxicaciones y la doble carga de la mala nutrición. Es particularmente preocupante en la niñez, donde estas condiciones han llevado incluso a la muerte infantil. Los riesgos a la salud, también se han observado en diferentes estados del país, como Colima ^23^, Sonora ^10^ y Michoacán ^24^.

Además, el idioma actúa como una barrera para poder acceder a la educación, para exigir sus derechos, comunicarse asertivamente y establecer una relación contractual con el empleador, debido a que gran parte de las personas jornaleras agrícolas con migración interna es monolingüe, y el sistema actual está constituido para hablantes del castellano, sin un enfoque intercultural. Destacamos cómo las redes de intermediarios perpetúan las inadecuadas condiciones laborales, agregado a que existe resistencia y secretismo para conocer los nombres de las empresas agroindustriales, situación que imposibilita la transparencia e identificación de actores en la cadena de producción y la exigencia de derechos, permitiendo que se sigan reproduciendo prácticas de dominación y brechas de poder. 

Otro aspecto relevante que deseamos resaltar es el papel de ser niña y mujer jornalera, situación que las coloca en una posición de desventaja. Desempeñan un doble rol: el de niñas cuidadoras o trabajadoras agrícolas, y el ser responsables de las labores domésticas y la alimentación familiar. Molina Rodríguez ^23^, señala cómo desde la niñez, se les imponen roles de género y subordinación. Además, debido a su condición de mujer jornalera, racializada, migrante, empobrecida y sin acceso a educación, las oportunidades de desarrollo son limitadas, lo que dificulta el pleno alcance de la dimensión de razonamiento.

Los resultados muestran cómo la migración temporal vulnera las condiciones de alimentación de las personas jornaleras agrícolas con migración interna, exponiéndolos a un ambiente alimentario obesogénico en la ruta migratoria. Sin embargo, en los lugares de destino, buscan replicar aspectos de su dieta tradicional, donde tienen cierto control sobre su alimentación, como la cría de aves y siembra para autoconsumo. Un ejemplo destacable es la crianza de guajolotes y gallinas que llevan consigo en los traslados, y que podría contribuir a mejorar su alimentación, como se ha visto en otros estudios ^25^. 

La ausencia del Estado y la desarticulación de las políticas dirigidas a persona jornalera agrícola con migración interna, se refleja en el ambiente alimentario institucional, en donde no existen las condiciones mínimas para su alimentación, como la designación de espacios para comer e insumos para la inocuidad alimentaria. A su vez, la accesibilidad y disponibilidad física a alimentos saludables resulta inasequible, ya que los campos están inmersos en “desiertos alimentarios” (8), que son aquellos espacios donde los alimentos frescos están a más de 1km, o “pantanos alimentarios” (2), donde las opciones de comestibles ultraprocesados sobrepasan las alternativas de alimentos saludables ^14^. Los mismos imposibilitan el acceso a una dieta saludable y el goce de salud. Black et al. ^26^, similarmente, encontraron que la población con mayor nivel de pobreza y de grupos étnicos son los que tienen una mayor oferta de comestibles ultraprocesados. De igual forma, estudios realizados en Sonora por Castañeda et al. ^13^, coinciden en que el trabajo temporal, condiciones de vida y situación económica hace que aumente la inseguridad alimentaria, causando daños a su salud. 

En su trabajo como jornaleros, al vivir con precariedad laboral y vulnerabilidad social, limita que puedan exigir condiciones laborales justas. La persona jornalera agrícola con migración interna no percibe la alimentación como un derecho o demanda prioritaria, sino como un aspecto secundario frente a otras necesidades. Esta percepción refleja una aceptación de condiciones laborales mínimas, lo cual limita sus expectativas y exigencias sobre aspectos básicos como la alimentación. Aranda & Castro Vásquez ^27^, coinciden que ocupan una posición débil en cuanto al capital hegemónico de la agroindustria y que su capacidad para generar cambios en las relaciones de poder en la estructura del campo es muy limitada. Sin embargo, se ha visto que la acción colectiva puede hacer frente a la demanda del cumplimiento de sus derechos, tal es el caso de la movilización de jornaleros en el valle de San Quintín ^28^.

A partir de la vulneración de las dimensiones de justicia social, el uso de prospectiva como herramienta para prever otros futuros, permitió elaborar propuestas desde la voz de actores clave para garantizar el derecho humano a la alimentación desde la soberanía alimentaria. A un corto plazo, se plantea que las personas jornaleras agrícolas con migración interna realicen migración interna bajo condiciones dignas y con el cumplimiento de sus derechos laborales. El ambiente alimentario institucional tendría que garantizar un espacio para consumir y guardar sus alimentos, horario de comida, predios de autoconsumo y traspatio, los cuales ya están establecidos en la Ley Federal del Trabajo ^29^.

No obstante, se deben hacer reformas que refuercen los mecanismos y monitoreo para su cumplimiento, con mayores sanciones para los empleadores que no cumplan con la ley, sin que se criminalice a las familias, sin corrupción y conflicto de interés con las empresas agroindustriales. La importancia de sancionar la producción de alimentos bajo condiciones de precariedad fue resaltada por los legisladores, así como el papel de la Secretaría del Trabajo y Previsión Social. 

Esto puede ser plausible, si se toman las experiencias exitosas en otros países como Argentina y Uruguay, quienes han logrado establecer políticas para aumentar la cobertura de la seguridad social. Igualmente, en Chile, a pesar de que comparte situaciones de precariedad laboral, tienen un mejor salario, una menor tasa de informalidad y el ambiente alimentario institucional garantiza aspectos para la alimentación durante la actividad laboral ^5^.

A partir del escenario ideal, se considera que las personas jornaleras agrícolas con migración interna no tendrían que migrar y deberían poder vivir dignamente en sus comunidades. En este sentido, las acciones a largo plazo deben considerar políticas públicas encaminadas en garantizarles el derecho humano a la alimentación como la reciente expedición de la Ley General Alimentación Saludable y Sostenible (LGAAS) ^30^ y su aplicación. Se deben diseñar e implementar estrategias locales, regionales y nacionales que vayan transitando hacia un cambio del modelo agrícola, que considere la autosuficiencia y soberanía alimentaria, y la movilización de la economía local. Se requiere de voluntad política, sinergia y articulación entre gobierno, organizaciones de la sociedad civil, academia y la acción colectiva de las personas jornaleras agrícolas con migración interna. Asimismo, es necesario un cambio en el paradigma alimentario en donde el alimento no sea visto como mercancía, para poder hacer frente al régimen agroalimentario actual ^2^.

Las políticas agroalimentarias en México deben considerar como elementos clave las condiciones laborales y los ambientes institucionales, que deben ser garantía de derechos desde la dignidad de las personas. Se exhorta a la conducción de futuras investigaciones similares en otros Estados para poder ampliar el panorama nacional. Asimismo, se deben enfocar esfuerzos en áreas claves para la salud pública, como la expedición e implementación de los lineamientos a la LGAAS. Además, de impulsar programas sociales que consideren las condiciones particulares de la población jornalera, ya que, por la falta de documentos, acceso a educación y migración, no cumplen con los requisitos de programas actuales como “Becas Benito Juárez” y “Sembrando Vida”. Lo que limita su acceso a derechos y apoyos fundamentales. 

La población jornalera migrante es un grupo diverso y varía por cada región, cultura, tipo de empleador, cultivo y destino de producción agrícola (mercado interno o de exportación), por lo que los resultados se limitan al contexto descripto. La migración temporal, sumada a la incertidumbre laboral y largas jornadas, fueron una limitante para realizar talleres y un mayor número de entrevistas con mujeres jornaleras. Sin embargo, la experiencia de la autora principal, quien ha trabajado con esta población por más de ocho años, así como la información proporcionada por otros informantes clave, fueron fundamentales para lograr un entendimiento profundo del contexto.

## Conclusiones 

De acuerdo con los resultados obtenidos, se pudo observar que las personas jornaleras agrícolas com migración interna experimentan vulnerabilidad por la migración forzada y la precariedad laboral, que se traduce en la transgresión de las seis dimensiones de justicia social e impide alcanzar el bienestar para una vida digna. Estas se ven reflejadas en un ambiente alimentario que promueve injusticias, desigualdad y vulnerabilidad alimentaria, particularmente en el ambiente del hogar, institucional y de abastecimiento, donde hay un limitado acceso a alimentos frescos y sanos, y que favorecen la adquisición de comestibles ultraprocesados (todas estas características del régimen alimentario neoliberal). Asimismo, la construcción de escenarios en términos prospectivos desde la voz de actores clave, permite proponer recomendaciones que ayuden a garantizar condiciones laborales dignas contemplando el derecho humano a la alimentación, con el fin caminar hacia un escenario ideal, donde la migración de las personas jornaleras agrícolas con migración interna sea una opción y no una obligación. Se refuerza la necesidad urgente de políticas públicas orientadas a mejorar las condiciones laborales, de vida y de alimentación en áreas rurales a través del impulso de las economías locales, que contemplen la soberanía alimentaria con enfoque intercultural, de género y la protección del interés superior de la niñez de las personas jornaleras agrícolas con migración interna que actualmente se encuentran en una posición de migración interna forzada.
